# Clinical simulation in health education: a systematic review*

**DOI:** 10.17533/udea.iee.v41n2e08

**Published:** 2023-08-24

**Authors:** Marta Alonso-Peña, Carmen Álvarez-Álvarez

**Affiliations:** 1 Ph.D. in Pathophysiology and Pharmacology. Postdoctoral researcher. Instituto de Investigación Sanitaria Marqués de Valdecilla -IDIVAL-, Spain. Email: martaalonsop@gmail.com. https://orcid.org/0000-0003-0934-2202 Instituto de Investigación Sanitaria Marqués de Valdecilla Spain martaalonsop@gmail.com; 2 Ph.D. in Pedagogy. Full Professor. Department of Education. Universidad de Cantabria, Spain. Email: alvarezmc@unican.es. https://orcid.org/0000-0002-8160-2286 Universidad de Cantabria Department of Education Universidad de Cantabria Spain alvarezmc@unican.es

**Keywords:** simulation training, educational technology, students, health occupations, entrenamiento simulado, tecnología educacional, estudiantes del área de la salud, treinamento por simulação, tecnología educacional, estudantes de ciências da saúde

## Abstract

**Objective:**

To summarize the most recent scientific evidence on the usefulness and implementation of simulation training programs for health science students.

**Methods:**

A search and systematic review were conducted of the literature through the use of the PRISMA guidelines using the terms MESH Simulation AND healthcare AND Professional Training, including 42 articles.

**Results:**

The bibliometric analysis revealed that most of the studies were local in nature, that is, conducted in a single center, or in a few centers in the same region, from the English-speaking world, and using a mixed methodology with pre/post-test measurements. As for the educational aspects, most of the studies were conducted at universities or in the area of continuous education, used multidisciplinary teams as the student target, and used role-playing games as the simulation method. Also, these programs were especially successful in the acquisition of competencies, such as teamwork, communication, and trust.

**Conclusion:**

Clinical simulation is a teaching methodology implemented in the last twenty years, mainly in English-speaking countries; it utilizes techniques for its execution and assessment that have been validated in contrasted in many scientific studies, and lastly, it was also observed that it is useful for providing training on general competencies for multidisciplinary groups.

## Resumen

**Objetivo:**

Resumir la evidencia científica más reciente sobre la utilidad e implementación de programas de formación mediante simulación en estudiantes de ciencias de la salud.

**Métodos:**

Se ha desarrollado una búsqueda y revisión sistemática de la literatura mediante la guía PRISMA empleando los términos MESH *Simulation* AND *healthcare* AND *Professional Training,* incluyéndose 42 artículos.

**Resultados:**

El análisis bibliométrico reveló que la mayoría de estudios eran de ámbito local, es decir, desarrollados en un único centro o en unos pocos centros de una misma localidad, procedentes del mundo anglosajón, y utilizaban una metodología mixta con pre/post-test. En cuanto a los aspectos educativos, la mayoría de estudios se desarrollaron a nivel universitario o en el ámbito de la formación continua, tuvieron como alumnado objetivo equipos multidisciplinares y utilizaron el juego de rol como método de simulación. Además, estos programas fueron especialmente exitosos en la adquisición de competencias como el trabajo en equipo, la comunicación y la confianza.

**Conclusión:**

La simulación clínica es una metodología docente que se ha ido implantando progresivamente durante las últimas dos décadas, mayoritariamente en países anglosajones, que utiliza técnicas para su ejecución y evaluación validadas y contrastadas en múltiples estudios científicos, y que resulta útil para el entrenamiento de competencias genéricas y equipos multidisciplinares.

## Resumo

**Objetivo:**

Resumir as evidências científicas mais recentes sobre a utilidade e implementação de programas de treinamento de simulação em estudantes de ciências da saúde.

**Métodos:**

Uma busca sistemática e revisão da literatura foi realizada usando o guia PRISMA usando os termos MESH *Simulation* AND *Healthcare* AND *Professional Training,* incluindo 42 artigos.

**Resultados:**

A análise bibliométrica revelou que a maioria dos estudos foram locais, ou seja, desenvolvidos num único centro ou em alguns centros de uma mesma cidade, do mundo anglo-saxão, e utilizaram uma metodologia mista com pré/pós- teste. Quanto aos aspectos educacionais, a maioria dos estudos foi realizada no nível universitário ou no campo da formação contínua, os alunos-alvo eram equipes multidisciplinares e usaram a dramatização como método de simulação. Além disso, esses programas foram especialmente bem-sucedidos na aquisição de habilidades como trabalho em equipe, comunicação e confiança.

**Conclusão:**

A simulação clínica é uma metodologia de ensino que tem vindo a ser progressivamente implementada ao longo das duas últimas décadas, maioritariamente em países anglo-saxões, que utiliza técnicas para a sua execução e avaliação validadas e contrastadas em múltiplos estudos científicos, e que é útil para o treino de competências genéricas. equipes multidisciplinares.

## Introduction

Simulation training is an experience-based teaching methodology for rehearsing events in a safe environment.^1^^,^^2^ The use of simulators in any area is based on two principles: guaranteeing safety and preventing critical errors.^3^ In the case of clinical simulation, the manufacturers of medical equipment were the first to promote its initial development,^4^^,^^5^ but during its evolution, more attention has been paid to the underlying pedagogy.^6^ All simulation programs follow a well-defined structure, with clear pedagogic objectives, and following a series of stages: (a) *Prebriefing*: an initial informational session in which guidance is provided to students on the objective of the simulation, the environment, and the tools that will be utilized.^4^ (b) Scenario: this is the simulation experience itself, designed in agreement with the learning objectives, in which the students will perform various procedures, and make decisions that are similar to real clinical contexts.^4^^,^^7^ (c) *Debriefing*: time dedicated for reflecting on the events that took place during the simulated situation. This is the moment in time in which to confront and discuss the errors, as well as the technical and cognitive skills of students.^3^^,^^8^ Experiential learning is acquired in the *debriefing* phase, thanks to the reflection performed on the experience itself.^9^ Thus, many simulation programs include various scenarios in which different students participate, while the rest become observers. Likewise, some programs record the development of the scenario to later make comments and discuss it in the *debriefing* phase. Given its importance, many specific tools and guides have been developed to structure the *debriefing*, such as “The Diamond”^9^ and “Promoting Excellence and Reflective Learning in Simulation” (PEARLS).^10^

The simulation scenario can be developed through different tools, which result in many different simulation methods. Thus, we can differentiate between scenarios based on role playing, in which the students enter a controlled physical space, and which can be classified as simulation with manikins or anatomical models^11^^,^^12^ and simulations with actors, standardized patients or role-playing;^13^ and methods based on Information and Communication Technologies (ICT),^12^ which can be sub-classified as computer-based simulations^14^ and virtual reality methods.^15^

An important aspect in the design of training programs based on simulation is fidelity, which refers to the degree in which the simulation reproduces reality.^12^ The degree of fidelity depends on many aspects, mainly the realism of the simulator, the equipment used, and the degree in which the students are able to overcome their disbelief and act in the simulation as if they would in the real world.^12^ Having this in mind, clinical simulations are classified as low, medium, and high fidelity, with the latter being the *gold standard* in the field of simulation.^16^ Recently, a step forward was been taken with *in situ* simulation programs. These simulations take place in the space in which real clinical activities take place, thereby allowing health professionals to practice their skills in the work environment itself.^17^

The general objective of the present study is to analyze the most recent scientific evidence on the usefulness and implementation of training programs through simulations for Health Professionals. The specific objectives are: (i) to describe the scientific literature in the field of clinical simulation as an education method; (ii) to discover the characteristics of the most-utilized simulation methods and their efficacy, and (iii) to study the degree of implementation of simulation as a teaching methodology in different areas of healthcare.

## Methods

For the development of the search and systematic review, the PRISMA (Preferred Reporting Items for Systematic Reviews and Meta-Analyses) method^18^ was used on large databases, *Web of Science* and *Scopus*, with the following combinations of keywords: (*Simulation* AND *healthcare* AND *Professional Training*) OR (Simulación AND Formación profesional). The inclusion criteria that the articles had to meet to be included in the review were: (a) complete original scientific articles, in English or Spanish, published in scientific journals, (b) articles published in the last 5 years, (c) articles that describe a simulation in the area of Health care, as well as a method of evaluation of its quality of training and the results of this evaluation, highlighting the pedagogic point of view, and (d) the study subjects must be Health Professionals, that is, health sciences students.

**Figure 1:**
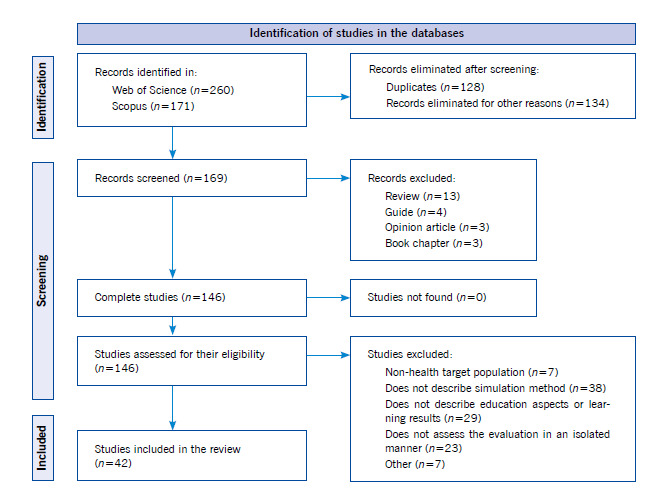
Summary of the articles identified in the systematic search, excluded and included in the review

The following filters were applied during the search: (a) the words selected must be found in the article’s abstracts, (b) articles published between 2017 and 2021, (c) original scientific articles published in scientific journals, (d) articles published in English or Spanish (Figure 1).

For the evaluation of the studies obtained, a registration and analysis template was designed. The variables for which a specific number of options were available, were categorized, while the others were maintained with empty fields, for a qualitative study. After the systematic reading of the articles and the collection of data, the quantitative variables were analyzed with the graphics and statistical package Excel 2016. The numerical variables are presented as mean and standard deviation (SD), while the categorical ones were analyzed based on their frequency, and are therefore presented as percentages (%) of the total or absolute value (*n*). A thematic analysis was performed with the qualitative variables.

## Results

### Bibliometric analysis

Most of the studies included were accepted in 2020.^19^^-^^24^^,^^25^^,^^26^^-^^31^ With respect to the geographical distribution of the studies, most of them were conducted in the United Kingdom (29%) and the United States (21%), and were local, that is, they were conducted in a single center, or at different centers in the same city (81%), no international studies or studies conducted in collaboration between more than one country were found, while only three were conducted at the national level.^19^^,^^20^^,^^32^ Only one article from a Spanish-speaking country was included (Argentina).^30^

### Methodological characteristics of the studies

A predominance of pre/post-test and mixed studies was observed. The most-utilized data collection instruments were: questionnaires (*n*=19) specifically designed by the research team, and composed by various types of questions, and externally-validated questionnaires (*n*=14 that assessed the students’ perceptions about their learning. The use of Likert-type scales was underlined in both types of questionnaires.

Within the qualitative studies (*n*=21), most used a thematic analysis of the data collected from one or many of the following sources: Open-ended questions included in the questionnaires (*n*=16); *Focus groups* or in-depth interviews (*n*=8), and *debriefing* sessions recorded on video (*n*=2).^32^^,^^33^ Some studies (*n*=15), also included data for the external assessment of the competencies acquired, such as the recording of events at hospitals, or assessments by their peers, the research team, or those in charge of the students, through the use of *checklists*, tests, and parameters collected by the instruments or manikins used. Among the main limitations of the studies, the most common aspects were related with the sample (small, convenience, low follow-up, previous experience with simulation, voluntary participants, or lack of a control group), and data collection (lack of validated instruments, data collection through the phone or online, lack of long-term data, and assessment of the impact of the program beyond the assessment by students).

### Characteristics of the students

Most of the articles analyzed (60%, *n*=25) presented proposals framed within non-formal education, more specifically, continuous education, as an improvement strategy for professional qualifications. The rest of the works had formal education proposals (40%, *n*=17), with all of them conducted at the university level. In this area, it is important to underline that 67% of the studies were directed to more than one target professional. In this group of articles, the objective in most of them (75%) was the training of interprofessional teams, that is, the target was a team of professionals, instead of a specific type of professional, underlining the need and importance of multidisciplinary collaboration in complex tasks. Likewise, seven studies were identified,^19^^,^^22^^,^^34^^-^^38^ whose objective was the training of diverse types of professionals, with nursing personnel represented in all of them.

### Methodological trends in simulation training

Most of the studies reviewed (64%) were only based on the role-playing methodology. On the other hand, about 15% of the works described a combination of more than one simulation method, with the role-playing one present in all of them, along with another method (computer simulation, virtual reality, and simulation with anatomical models). Only 17% (*n*=7) of the studies presented proposals that included the use of simulations via computer or virtual reality, and within them, almost half (*n*=3) were combined with role-playing.^30^^,^^34^^,^^39^ In most of them, the *prebriefing*, scenarios, and *debriefing* structure was followed. More specifically, nine articles used structured or validated *debriefing* models.^22^^,^^31^^,^^35^^,^^40^^-^^45^

As for fidelity, most of the article had high-fidelity simulation proposals (67%), while in 21% (*n*-9) of the articles, the type of fidelity could not be determined, as it was not specified in the text.^2^^,^^24^^,^^27^^,^^33^^,^^38^^,^^45^^-^^48^ With respect to the locations selected for the simulation to take place, 31% were conducted in simulation centers, and 24% at universities. It must be underlined that up to 19% (*n*=8) of the studies described simulation projects that took place *in situ*.^2^^,^^28^^,^^31^^,^^38^^,^^49^^-^^52^ As for the types of competencies that were worked on in the different simulation proposals, most of the studies included the acquisition of general or cross-sectional competencies, either in combination with the acquisition of specific competencies (43%, *n*=18), as well as in an isolated manner (36%, *n*=15). Thus, teaching methods with simulation seem to be evolving from technical competencies, to general ones. More specifically, three works^33^^,^^34^^,^^53^ aimed at training students on the use of the communication tool SBAR (*Situation, Background, Assessment and Recommendation*).^54^ This tool is becoming important in the area of health, for communication between professionals and patients. Also, in two studies,^34^^,^^47^ the simulation consisted in students experiencing the point of view of the patient and the complexity of the procedures they are subjected to, to stimulate empathy with people who are sick.

### Results of learning through simulation

Most (*n*=32) of the studies described positive results in their objectives and hypotheses. Ten articles showed partially negative results with respect to their learning objectives. The negative results were associated with: (a) Simulation proposals based on ICT: one of them was not effective,^20^ while the bad execution of the rest was due to a connection problems, software, or the lack of awareness about the importance of learning through the use of these technologies.^34^ (b) Acquisition of knowledge: two studies showed the same efficacy between presentation-based methods, or conventional independent work,^29^^,^^55^ while in others, no differences were observed in this specific area between the pre- and post-test. (c) Target students: in the studies with groups of heterogeneous professionals, some of the learning results were not met in certain types of professionals, although they were obtained in the group of students as whole. The studies with a long-term assessment observed a decrease in the knowledge or skills acquired, which indicates the need for constant updating in this area, and justifies the planning of repeated training sessions.

The results of the qualitative analyses from most of the studies stressed improvements in: leadership and communication, teamwork, critical thinking, reflective learning, making of decisions, trust, and clinical skills. It is important to note that in all the studies that assessed the perception of the students on the simulation methodology, the responses were favorable towards the use of this training method, and the importance of learning by doing. In one study, only a minority of the students indicated their preference for presentation-type methods, as compared to computer-based simulations.^55^ On the other hand, the thematic analysis of a study whose simulation proposal was developed at a large scale, also revealed the theme of the complexity in organizing and developing a simulation with these characteristics.^28^ Additionally, the intervention developed in four studies promoted the assessment of errors and risks, and changed the management of the medical services themselves, where the interventions took place.^31^^,^^49^^-^^51^

## Discussion

There is a large body of scientific bibliography on the field of simulation as a teaching-learning method for Health Professionals. However, publication in high-impact scientific journals predominated in English-speaking countries, and were immersed in university education and continuous education. Teaching through simulation is a well validated methodology.^2^ It has repeatedly been shown that it improves competency in many skills,^56^ as well as the trust perceived, the behaviors of speaking out loud, communication, and teamwork,^57^ so that simulation as an teaching method is deemed to be an excellent tool for addressing the skills required in multidisciplinary teams.^56^ The results of this systematic review indicate that the simulation methods did not increase the level of knowledge acquired, although they benefited the acquisition of skills and competencies, the central axes of modern education.^58^ Most of the works analyzed showed that these skills were acquired in a more significant manner in the high-fidelity simulation method through role-playing, in which person-to-person interactions are produced in a space with high-fidelity. The posterior *debriefing* sessions stimulate self-criticism, supported or not by watching the recorded scenarios, so that this methodology successfully combines experiential and reflective learning.^59^ Likewise, most of the simulation programs were positively evaluated by students, although only a few studies assessed the efficacy of this methodology beyond the first evaluation model by Kirkpatrick.^60^

On the other hand, although simulation through the use of virtual reality seems to be a very attractive area in the field of health education,^15^ the present review indicates that currently, there is little evidence on this respect. It is perhaps that this development is hampered by the technological difficulty inherent in simulation programs through virtual reality, together with its associated costs. Thus, the methods based on role-playing are still the *gold standard* in Healthcare. Also, the design of simulation programs must consider the need to repeat it in the long term, as our analysis pointed to the existence of a decrease in the knowledge or skills acquired through time; the modification of programs at the educational level and the training of the students, especially in the case of interdisciplinary groups, to obtain good results ;^61^ or the costs and logistic complications derived from the organization of large simulation training programs.^28^

Among the limitations of the study, we must consider those from the included studies themselves. Most of the studies were local in nature, and with small samples selected by convenience. However, most of the studies included utilized a mixed methodology and assessed the learning of the individuals through repeated measurements before and after the event (pre/post-test), and their systematic analysis offered very homogeneous results with respect to the advantages of learning through simulation.^4^

In conclusion, clinical simulation is a teaching methodology that has been progressively implemented in the last two decades, mostly in English-speaking countries, that utilizes techniques for its execution and assessment that have been validated and contrasted in many scientific studies, and which is useful for the training of general competencies and multidisciplinary teams.

## References

[1] Good ML. (2003). Patient simulation for training basic and advanced clinical skills. Med. Educ..

[2] Maenhout G, Billiet V, Sijmons M, Beeckman D (2021). The effect of repeated high-fidelity in situ simulation-based training on self-efficacy, self-perceived leadership qualities and team performance: A quasi-experimental study in a NICU-setting. Nurse Educ. Today..

[3] López M, Ramos L, Pato O, López S (2013). La simulación clínica como herramienta de aprendizaje. Cir. Mayor Ambulatoria..

[4] Saiz Linares Á. (2012). La simulación clínica y la formación de profesionales reflexivos: dos experiencias en la formación inicial de médicos.

[5] Bienstock J, Heuer A (2022). A review on the evolution of simulation-based training to help build a safer future. Medicine (Baltimore).

[6] Hallmark B, Brown M, Peterson DT, Fey M, Decker S, Wells-Beede E (2021). Healthcare Simulation Standards of Best PracticeTM Professional Development. Clin. Simul. Nurs..

[7] Backlund P, Maurin Söderholm H, Engström H, Andersson Hagiwara M, Lebram M (2018). Breaking Out of the Bubble Putting Simulation Into Context to Increase Immersion and Performance. Simul. Gaming..

[8] Aitken JA, Torres EM, Kaplan S, DiazGranados D, Su L, Parker S (2021). Influence of simulation-based training on reflective practice. BMJ Simula. Tech. Enhanc. Learn..

[9] Jaye P, Thomas L, Reedy G (2015). 'The Diamond': a structure for simulation debrief. Clin Teach..

[10] Eppich W, Cheng A (2015). Promoting Excellence and Reflective Learning in Simulation (PEARLS): development and rationale for a blended approach to health care simulation debriefing. Simul. Healthc..

[11] Ayaz O, Ismail FW (2022). Healthcare Simulation: A Key to the Future of Medical Education - A Review. Adv.Med. Educ. Pract..

[12] Brindley PG, Suen GI, Drummond J (2007). Part two: Medical simulation. how to build a successful and long-lasting program. Can. J. Respir. Ther..

[13] Nestel D, Tierney T (2007). Role-play for medical students learning about communication: guidelines for maximising benefits. BMC Med. Educ..

[14] Romero Lopez D, de Benito Crosetti B (2020). Diseño de una propuesta didáctica para el uso de simuladores virtuales en la rama sanitaria de Formación Profesional. RIITE Rev. Interuniversitaria Invest. Tecnol. Educ..

[15] Bracq MS, Michinov E, Jannin P (2019). Virtual Reality Simulation in Nontechnical Skills Training for Healthcare Professionals: A Systematic Review. Simul Healthc..

[16] Dávila-Cervantes A. (2014). Simulación en Educación Médica. Investigación en Educación Médica.

[17] Martin A, Cross S, Attoe C (2020). The Use of in situ Simulation in Healthcare Education: Current Perspectives. Adv.Med. Educ. Pract..

[18] Page MJ, McKenzie JE, Bossuyt PM, Boutron I, Hoffmann TC, Mulrow CD (2021). The PRISMA 2020 statement: an updated guideline for reporting systematic reviews. BMJ..

[19] Farias Da Guarda SN, Santos JPS, Reis MSM, Da Hora Passos R, Correia LC, Caldas JR (2021). Realistic simulation is associated with healthcare professionals’ increased self‑perception of confidence in providing acute stroke care: A before‑after controlled study. Arquiv. NeuroPsiquiatr..

[20] Yeo CL, Ho SKY, Tagamolila VC, Arunachalam S, Bharadwaj SS, Poon WB (2020). Use of web-based game in neonatal resuscitation - Is it effective?. BMC Medical Education.

[21] Yu J, Lee W, Kim M, Choi S, Lee S, Kim S (2020). Effectiveness of simulation-based interprofessional education for medical and nursing students in South Korea: a pre-post survey. BMC Med. Educ..

[22] MacCuish AH, McNulty M, Bryant C, Deaner A, Birns J (2021). Simulation training for clinicians returning to practice. Br. J. Hosp. Med..

[23] Wheeler M, Powell E, Pallmann P (2021). Use of High-fidelity simulation training for radiology healthcare professionals in the management of acute medical emergencies. Brit. J. Radiol..

[24] Harden A, Ragoonanan D, Anildes-Gubman D, McCall D, Faltus K, Featherston S (2020). Chimeric Antigen Receptor, Teamwork, Education, Assessment, and Management (CAR-TEAM): A Simulation-Based Inter-professional Education (IPE) Intervention for Management of CAR Toxicities. Front. Oncol..

[25] Hinduja A, Rishipathak P, Vijayaraghavan S (2020). Impact of high fidelity simulation training on the quality of cardiopulmonary resuscitation performance among emergency medical services (Ems) professionals in Pune, India. Indian J. Forensic Med. Toxicol..

[26] Spence H, Somasundram K, Biyani CS, Jain S (2020). Training Nontechnical Skills in Ward Rounds to Improve Team Performance. J. Surg. Educ..

[27] Giordano NA, Whitney CE, Axson SA, Cassidy K, Rosado E, Hoyt-Brennan AM (2020). A pilot study to compare virtual reality to hybrid simulation for opioid-related overdose and naloxone training. Nurse Educ. Today..

[28] Saaranen T, Silén-Lipponen M, Palkolahti M, Mönkkönen K, Tiihonen M, Sormunen M (2020). Interprofessional learning in social and health care-Learning experiences from large-group simulation in Finland. Nurs. Open..

[29] Thompson J, White S, Chapman S (2020). Virtual patients as a tool for training preregistration pharmacists and increasing their preparedness to practice: A qualitative study. PLoS ONE..

[30] Cobián JI, Ferrero F, Alonso MP, Fontana AM (2021). How to train a complex skill in surgery: Qualitative evaluation of a simulation-based strategy. Rev. Argent. Cir..

[31] Sharara-Chami R, Sabouneh R, Zeineddine R, Banat R, Fayad J, Lakissian Z (2020). In Situ Simulation: An Essential Tool for Safe Preparedness for the COVID-19 Pandemic. Simul. Healthcare..

[32] Brezis M, Lahat Y, Frankel M, Rubinov A, Bohm D, Cohen MJ (2017). What can we learn from simulation-based training to improve skills for end-of-life care? Insights from a national project in Israel. Israel J. Health Pol. Res..

[33] Cunningham S, Musick DW, Trinkle DB (2021). Evaluation of an interprofessional learning experience for telephone consultations. Adv. Med. Educ. Pract..

[34] Aicken C, Hodgson L, de Vries K, Wilkinson I, Aldridge Z, Galvin K (2021). ‘This Adds Another Perspective’: Qualitative Descriptive Study Evaluating Simulation-Based Training for Health Care Assistants, to Enhance the Quality of Care in Nursing Homes. Int. J. Environ. Res. Public Health..

[35] Babu MV, Arumugam MK, Debnath DJ (2021). Simulated patient environment: A training tool for healthcare professionals in COVID-19 era. Adv. Med. Educ. Pract..

[36] James LS, Williams ML, Camel SP, Slagle P (2021). Nursing student's attitudes toward teams in an undergraduate interprofessional mass casualty simulation. Nurs. Forum..

[37] Santos TM, Pedrosa RBS, Carvalho DRS, Franco MH, Silva JLG, Franci D (2021). Implementing healthcare professionals’ training during COVID-19: A pre and post-test design for simulation training. Sao Paulo Med. J..

[38] Schram A, Paltved C, Christensen KB, Kjaergaard-Andersen G, Jensen HI, Kristensen S (2021). Patient safety culture improves during an in situ simulation intervention: A repeated cross-sectional intervention study at two hospital sites. BMJ Open Qual..

[39] Sapkaroski D, Baird M, Mundy M, Dimmock MR (2019). Quantification of Student Radiographic Patient Positioning Using an Immersive Virtual Reality Simulation. Simul. Healthcare..

[40] Attoe C, Lavelle M, Sherwali S, Rimes K, Jabur Z (2019). Student interprofessional mental health simulation (SIMHS): evaluating the impact on medical and nursing students, and clinical psychology trainees. J. Ment. Health Train Educa Pract..

[41] Kowalski C, Attoe C, Ekdawi I, Parry C, Phillips S, Cross S (2018). Interprofessional Simulation Training to Promote Working With Families and Networks in Mental Health Services. Acad. Psychiatry..

[42] Lavelle M, Abthorpe J, Simpson T, Reedy G, Little F, Banerjee A (2018). MBRRACE in simulation: an evaluation of a multi-disciplinary simulation training for medical emergencies in obstetrics (MEmO). J. Obstetr. Gynaecol..

[43] Lavelle M, Attoe C, Tritschler C, Cross S (2017). Managing medical emergencies in mental health settings using an interprofessional in-situ simulation training programme: A mixed methods evaluation study. Nurse Educ. Today..

[44] Piette AE, Attoe C, Humphreys R, Cross S, Kowalski C (2018). Interprofessional simulation training for community mental health teams: Findings from a mixed methods study. J. Interprof. Care..

[45] Quick C. (2022). Mandated Reporter Training for Pediatric Nurse Practitioner Students Using Virtual Simulation and Community Collaboration: A Pilot Event. Clin. Simul. Nurs..

[46] Blumling A, Kameg K, Cline T, Szpak J, Koller C (2018). Evaluation of a standardized patient simulation on undergraduate nursing students' knowledge and confidence pertaining to intimate partner violence. J. Forensic Nurs..

[47] Yang S, Chaudhary Z, Mylopoulos M, Hashmi R, Kwok Y, Colman S (2019). Using simulation to explore medical students' understanding of integrated care within geriatrics. BMC Medical Education.

[48] Amini H, Gregory ME, Abrams MA, Luna J, Roland M, Sova LN (2021). Feasibility and usability study of a pilot immersive virtual reality-based empathy training for dental providers. J. Dent. Educ..

[49] Shrestha R, Shrestha AP, Shrestha SK, Basnet S, Pradhan A (2019). Interdisciplinary in situ simulation-based medical education in the emergency department of a teaching hospital in Nepal. Int. J. Emerg. Med..

[50] Dochez V, Beringue F, Legendre G, Jeanneteau P, Rolland D, Coutin AS (2021). Assessment of a multiprofessional training programme by in situ simulation in the maternity units of the Pays de Loire regional perinatal network. J. Gynecol. Obstetr. Hum. Reprod..

[51] Kumar A, Sturrock S, Wallace EM, Nestel D, Lucey D, Stoyles S (2018). Evaluation of learning from Practical Obstetric Multi-Professional Training and its impact on patient outcomes in Australia using Kirkpatrick's framework: A mixed methods study. BMJ Open..

[52] Villemure C, Georgescu LM, Tanoubi I, Dubé JN, Chiocchio F, Houle J (2019). Examining perceptions from in situ simulation-based training on interprofessional collaboration during crisis event management in post-anesthesia care. J. Interprof. Care..

[53] Acharya R, Thomas G, Hellaby M (2017). Evaluation of a clinical handover simulation training session for junior doctors in psychiatry. BMJ Simul.Technol. Enhanc. Learn..

[54] Shahid S, Thomas S (2018). Background, Assessment, Recommendation (SBAR) Communication Tool for Handoff in Health Care - A Narrative Review. Saf. Health..

[55] Mai CW, Lee EL, Wong PS, Er HM (2019). Evaluation of computer-based simulation learning on knowledge, learning approaches and motivation among pharmacy students. Indian J. Pharm. Educ. Res..

[56] Naik VN, Brien SE (2013). Review article: simulation: a means to address and improve patient safety. Can J. Anaesth..

[57] Raemer DB, Kolbe M, Minehart RD, Rudolph JW, Pian-Smith MC (2016). Improving Anesthesiologists' Ability to Speak Up in the Operating Room: A Randomized Controlled Experiment of a Simulation-Based Intervention and a Qualitative Analysis of Hurdles and Enablers. Acad. Med..

[58] Aguerrondo I. (2009). Conocimiento complejo y competencias educativas. IBE/Unesco Working Papers In Curriculum Issues.

[59] Piña-Jimenez I, Amador-Aguilar R (2015). La enseñanza de la enfermeria con simuladores, consideraciones tecnico-pedagagógicas para perfilar un modelo didactico. Enferm. Univ..

[60] Kirkpatrick DL. (1994). Evaluating training programs: the four levels Four Levels of Training Evaluation.

[61] Weil A, Weldon SM, Kronfli M, Watkins B, Kneebone R, Bello F (2018). A new approach to multi-professional end of life care training using a sequential simulation (SqS Simulation™) design: A mixed methods study. Nurse Educa. Today..

